# The deubiquitinating enzyme USP17 is associated with nonsmall cell lung cancer (NSCLC) recurrence and metastasis

**DOI:** 10.18632/oncotarget.1282

**Published:** 2013-10-03

**Authors:** Cheryl McFarlane, Suzanne McFarlane, Ian Paul, Kenneth Arthur, Michael Scheaff, Keith Kerr, Michael Stevenson, Dean A. Fennell, James A. Johnston

**Affiliations:** ^1^ Centre for Infection and Immunity, Queen's University Belfast, Belfast, Northern Ireland, UK; ^2^ Centre of Cancer Research and Cell Biology, School of Medicine, Dentistry and Biomedical Sciences, Faculty of Medicine, Health and Life Sciences, Queen's University Belfast, Belfast, Northern Ireland, UK; ^3^ Department of Pathology, Barts & London NHS Trust, London, UK; ^4^ Department of Pathology, University of Aberdeen, Scotland, UK; ^5^ Centre for Public Health, School of Medicine, Dentistry and Biomedical Sciences, Faculty of Medicine, Health and Life Sciences, Queen's University Belfast, Belfast, Northern Ireland, UK; ^6^ Current address: University of Leicester & Leicester University Hospitals, MRC Toxicology Unit, Hodgkin Building, Leicester; ^7^ Current address: Amgen, One Amgen Center Dr., Thousand Oaks, CA

## Abstract

USP17 is a cell cycle regulated deubiquitinating enzyme that is highly expressed in tumor-derived cell lines and has an established role in cell proliferation and chemotaxis. This is the first study to examine the clinical significance of USP17 expression in non-small cell lung cancer (NSCLC). USP17 was overexpressed in both squamous and adenocarcinoma NSCLC tissue. Patients with USP17 positive tumors had significantly reduced recurrence-free survival than patients with USP17 negative tumors. Moreover, USP17 was more highly expressed in patients with recurrence of disease at distant sites, suggesting that USP17 levels may correlate with NSCLC distant metastases. Overall, these findings establish USP17 as a potentially valuable novel biomarker for metastatic lung cancer.

## INTRODUCTION

Ubiquitination is widely recognised as a fundamental regulator of numerous cellular events, indeed the role of ubiquitination in DNA damage response, cell cycle regulation and intracellular trafficking has been extensively characterised. Consequently, much attention has been focused on determining the association between ubiquitination and tumorigenesis [1]. However, in a manner akin to the relationship between phosphorylation and dephosphorylation, ubiquitination is a reversible process. The deconjugation of ubiquitin is catalysed by deubiquitinating enzymes (DUBs), which are emerging as equally important for cell function and tumorgenesis [2-4].

DUBs are emerging as potential anti-cancer targets due to their oncogenic and tumor suppressive functions [5]. The ubiquitin specific protease (USP) family are the largest and best characterised family of DUBs, consisting of over 60 members [6]. The DUB, USP7, is a component of the DNA damage checkpoint which deubiquitinates p53 and Mdm2 to alter the stability and activity of p53 and is differentially expressed in non-small cell lung cancer [RW.ERROR - Unable to parse:&nbsp;&nbsp;}} {{142Pei,D.2012]. This DUB also regulates the subcellular localization of the tumor suppressor PTEN. USP7 is overexpressed in prostate cancer where it drives the deubiquitination and nuclear exclusion of PTEN, which has been correlated with more aggressive disease [9]. Indeed, USP2a is overexpressed in prostate cancer and correlates with tumor progression and poor prognosis in oral squamous cell carcinoma [10-12]. Conversely, USP10 is thought to function as a tumor suppressor as it deubiquitinates and stabilises p53 in response to DNA damage and is downregulated in renal cell carcinoma where it correlates with reduced wild type p53 [13]. High expression levels of USP28 have also been reported in colon and breast carcinoma, the deubiquitinating activity of USP28 is known to stabilize the levels of the oncogenic transcription factor Myc however the clinical implications of this are currently unclear [14].

Recently, a global DUB study reported differential expression of many DUBs in a range of cancers [15]. Despite these compelling findings to suggest that DUBs are potentially novel oncogenes or tumor suppressors, there are few reports examining the association of DUBs with patient outcome. To date approximately 95 human DUBS have been reported however the specific substrates of many DUBs are yet to be identified, suggesting that much work will be required to understand their function and clinical significance [4, 16].

We identified a member of the USP superfamily, Ubiquitin Specific Protease 17 (USP17) and found that it regulated signalling through the Ras pathway by controlling the intracellular localisation of Ras and other small GTPases; key regulators of cell proliferation and migration [17-19]. Moreover, we determined that USP17 was differentially expressed during the cell cycle and discovered that USP17-knockdown caused a G1 cell cycle block and inhibited proliferation of tumor-derived cell lines by attenuating GTPase signalling [20]. In addition, we later demonstrated that USP17 expression was also upregulated by chemokines and that USP17-silencing blocked chemotaxis [21]. These findings indicated that USP17 potentially promoted tumor growth and metastasis yet USP17 had never been examined in a clinical context. In this study we examine the expression of USP17 in non-small cell lung cancer (NSCLC) tumors. Here we report that USP17 was differentially expressed in NSCLC tissue; specifically, USP17 was upregulated in squamous NSCLC patients in comparison to those with adenocarcinoma yet neither USP17 nor histology was associated with NSCLC prognosis. However patients with USP17 positive tumors had significantly reduced recurrence-free survival than patients with USP17 negative tumors. Moreover, USP17 correlated with the recurrence of disease at distant sites. These findings establish for the first time that USP17 may be a valuable as novel biomarker for metastatic lung cancer.

## RESULTS

### Clinicopathological Features of NSCLC cohort

The NSCLC cohort consisted of resected biopsy tissue from 100 patients with stage IA to IV disease. The main clinical and pathologic characteristics of the cohort are summarized in Table 1. The histology of the NSCLC cohort comprised of 71% squamous cell carcinoma and 29% adenocarcinoma which are representative of NSCLC histology incidence. The vast majority of the cohort did not receive neoadjuvant therapy (97%), and were primarily treated by surgical resection, 19% of which also received adjuvant therapy. In addition, 44% of patients received radiotherapy. Linked 8-year clinical follow up data was also available for this cohort that included detailed information regarding prognosis, disease recurrence and metastases.

**Table 1:** Clinicopathological Details of NSCLC cohort

Feature		Number of patients (n=100)
**Gender**	Male Female	69 31
**Age**	Mean +/− SD Median	65 +/− 9.1 67
**Histology**	T1N0M0 (IA) T2N0M0 (IB) T2N1M0 (IIB) T2N2M0 (IIIA) T3N0M0 (IIB) T3N1M0 (IIIA) T3N2M0 (IIIA) T4/AnyN/M0 (IIIB) Any T/N/M1 (IV)	15 31 11 16 12 6 2 4 3
**Tumor Location**	Right sided Left sided	61 39
**Neoadjuvant Therapy**	Yes No	3 97
**Adjuvant Therapy**	Yes No	19 81
**Radiotherapy**	Palliative Radical	28 16
**Surgical Procedure**	Lubectomy Pneumonectomy Wedge	71 19 10
**Cancer Recurrence**	Unknown Local/No recurrence Distant recurrence	3 55 42

### Differential expression of USP17 and NSCLC Histology

**Figure 1:**
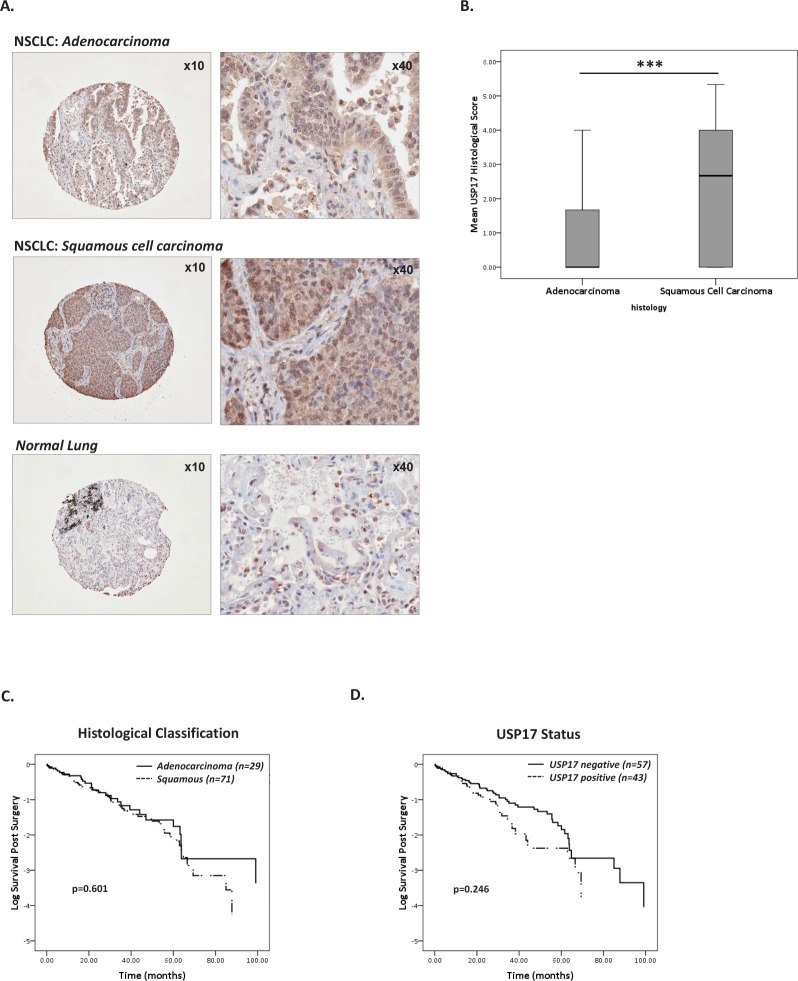
Characterisation of USP17 expression in NSCLC tissue (A) Representative images (magnification x10 & x40) of USP17 immunohistological staining of NSCLC adenocarcinoma (upper panel), squamous cell carcinoma (middle panel) and control non-neoplastic lung tissue (lower panel). (B) Box and whisker plots comparing the mean USP17 histological scores between adenocarcinoma and squamous cell carcinoma tissue. Squamous cell carcinoma tissue expressed significantly higher levels of USP17 than adenocarcinoma tissue (***p<0.001, t-test). (C) Kaplan Meier curves representing survival of NSCLC cohort when stratified according to tumor histology (adenocarcinoma n=29 & squamous cell carcinoma n=71). Histological subtype did not affect patient survival (p=0.601, log ranks test). (D) Kaplan Meier curves comparing patient survival when the cohort was stratified according to USP17 positivity (USP17 positive, n=43 and USP17 negative, n=57). USP17 levels were not associated with NSCLC patient survival (p=0.246, log ranks test).

We have previously reported that USP17 is highly expressed in a range of tumor biopsy samples including squamous NSCLC whilst negligible levels were detected in the corresponding non-neoplastic tissue[20]. However the clinical implications of this observation remained unknown. Consistent with our previous report, USP17 was highly expressed in NSCLC tissue and was not detected in matched non-malignant lung tissue. Histological scoring of USP17 levels revealed that 43% of the biopsies were positive for USP17. Furthermore, USP17 was differentially expressed in NSCLC biopsies depending on tumor histology (Figure 1a). Significantly higher levels of USP17 were detected in tumor tissue with squamous cell histology in comparison to adenocarcinoma biopsy tissue (p<0.001, Figure 1b).

### USP17 and NSCLC Overall Survival

Tumors were given a histological score for USP17 expression as either zero or ranging from 2-6 according to the proportion and intensity of staining. Patients were subsequently classified as positive (≥2) or negative (<2) for USP17 expression based on mean histological scores from replicate cores and statistically analyzes were carried out to correlate USP17 expression with the linked clinical data. No correlation was observed between USP17 and tumor stage, grade or treatment strategy. Indeed the relationship between USP17 and NSCLC histology was the only significant relationship identified. We next sought to examine how these factors where associated with patient survival post-surgery. There are conflicting reports regarding the correlation between histological subtype and NSCLC prognosis [22]. The mean overall survival time of patients in this cohort was 28.5±2.6 months post-surgery and NSCLC histology was not associated with prognosis in this cohort (Figure 1c; Squamous, mean survival 27.8 months; Adenocarcinoma, mean survival 31.3 months; p=0.601). To determine the prognostic value of USP17, Kaplan-Meier survival analysis was carried out to compare the USP17 positive and negative patients. Whilst an overall a trend of poorer prognosis was observed in the USP17-positive patient group (23.6±3.6 months) compared to the USP17-negative biopsies (31.0 ±3.5 months), this trend was not statistically significant (p=0.246, Figure 1d). Cox proportional hazards analysis confirmed that the combined impact of USP17 and histology had no bearing on overall survival (data not shown).

### USP17 correlates with reduced recurrence-free survival and distant metastasis

**Figure 2:**
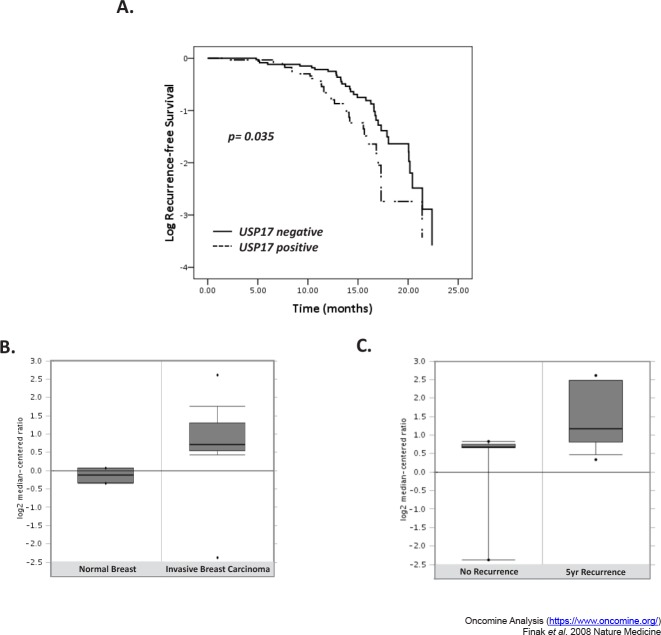
High USP17 levels correlated with reduced recurrence-free survival (A) Kaplan Meier curves comparing recurrence-free survival of patients when stratified according to USP17 positivity. The cohort was classified as either positive or negative for USP17 expression. The group with USP17 positive tumors had significantly earlier recurrence of disease than those with USP17 negative tumors (p=0.035, log ranks test). (B) Box and whisker plots illustrating USP17 expression levels in normal breast compared to invasive breast carcinoma tissue carried out by Oncomine analyses of the Finak *et al.* gene expression and predictive outcome in breast cancer study [23]. USP17 expression was higher in tumor tissue in comparison to non-neoplastic tissue. (C) Box and whistler plots depicting USP17 expression levels in patients with breast cancer recurrence within 5 years as compared to those with no disease recurrence.

In addition to information regarding overall survival we also had follow-up data detailing the period prior to disease recurrence. Kaplan Meier analysis was carried out to assess the correlation between USP17 and recurrence-free survival (Figure 2). Recurrence of disease occurred on average 13.67±1.1 months following diagnosis. The USP17-negative group had a significantly longer period of recurrence-free survival (mean 14.75±1.6 months) in comparison to the USP17 positive group (mean 12.42±1.6 months, p=0.035, Figure 2A). Histological subtype was not associated with recurrence-free survival (data not shown), indeed Cox proportional hazard analysis revealed that USP17 was the only parameter that was statistical associated with recurrence-free survival. The relationship between USP17 and NSCLC recurrence was corroborated by Oncomine analysis of the Finak *et al.* gene expression and predictive outcome in breast cancer study [23]. USP17 was expressed two-fold higher in breast carcinoma tissue in comparison to normal breast tissue (Figure 2B). Moreover, USP17 was more highly expressed in patients that had breast cancer recurrence within 5 years as compared to patients that had no disease recurrence (Figure 2C).

**Figure 3:**
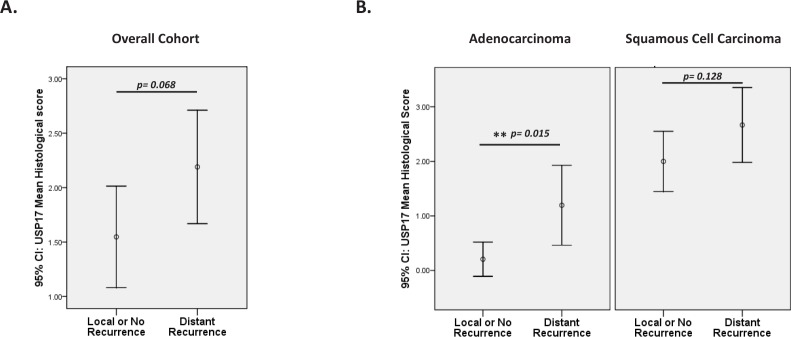
USP17 and distant metastases of NSCLC (A) Patients were categorised into two groups according to the site of disease recurrence; either those with no or local recurrence compared to those with metastatic NSCLC whose disease had recurred at distant sites. USP17 was higher in patients with metastatic disease but this was not statistically significant (p=0.068, t-test) (B) Patients were grouped according to histology (adenocarcinoma, left panel and squamous cell carcinoma, right panel) and levels of USP17 were compared between those with no/local recurrence of disease to those with metastatic disease. USP17 was expressed at significantly higher levels in the adenocarcinoma patients those disease had metastasised to distant sites (p=0.015, t-test).

In addition to recurrence data, there was also detailed information documenting the sites of disease recurrence. This enabled the distinction to be made between patients that had local recurrence of disease from those whose disease had metastasised to distant sites, most commonly the brain, bones and liver. Patients were classified into one of two categories according to disease recurrence; those with either no/local recurrence (55%) or those with distant recurrence (42%) (Table 1). The relationship between USP17 and the sites of disease recurrence were also examined. The mean expression levels of USP17 were compared between the recurrence groups. In general, USP17 was more highly expressed in patients with distant recurrence yet this did not reach statistical significance (Figure 3a). However, on the basis that USP17 correlated strongly with the histological classification of NSCLC (Figure 1), the cohort was stratified according to histology and the analysis repeated. In the adenocarcinoma group, USP17 levels were significantly higher in the patients with recurrence of disease at distant sites (Figure 3b, left panel). Indeed USP17 levels significantly correlated with metastasis of NSCLC adenocarcinoma (Spearmans correlation p=0.021). USP17 was globally higher in the squamous group, those with distant recurrence also tended to have elevated levels of USP17 although this trend did not reach statistical significance (Figure 3b, right panel). These findings revealed a novel relationship between USP17 expression and early recurrence of disease and the metastasis of NSCLC to distant sites.

## DISCUSSION

Following the recent publication of seminal studies identifying the mechanistic role of USP17 in the control of cell division and migration, this study presents the first examination of USP17's clinical relevance [20, 21]. One of the most striking findings of this study revealed that USP17 was expressed at significantly higher levels in squamous tissue in comparison to adenocarcinoma tissue. The underlying mechanism accounting for differences in USP17 levels between NSCLC histology is yet unclear. Molecular profiling of NSCLC histological subtypes has revealed several features which distinguish adenocarcinoma from squamous cell carcinoma. Most notably squamous cell carcinomas are mostly commonly associated with smoking history and are marked by amplification of PIK3CA and p63; whereas adenocarcinomas are associated with a high frequency of mutations in KRas and EGFR [24, 25]. The mechanism driving increased expression of USP17 in squamous cell carcinoma in comparison to adenocarcinoma are yet unclear and may arise due to differences in transcriptional regulation or protein turnover. Moreover, the clinical implications of the differential USP17 between histological subtypes is also unclear as neither USP17 nor histological subtype was associated with overall patient prognosis in this cohort.

We have previously reported that USP17 expression was induced by a range of chemokines and coordinates the chemotaxis and invasion of tumor-derived cell lines *in vitro*, suggesting that USP17 could potentially promote tumor cell metastasis [21]. This hypothesis is corroborated by our current observations. USP17-positive NSCLC tumors were associated with reduced recurrence-free survival; USP17 was also elevated in patients with 5-year recurrence of breast cancer, suggesting that USP17 may function as a marker of early disease recurrence. Moreover, by categorising the patients into those that subsequently developed either local or distant cancer recurrence we were able to demonstrate that USP17 levels were higher in NSCLC patients whose disease had metastasised to distant sites including the adrenal glands, liver, brain, and bone. Overall, these findings suggest that this DUB is a potentially novel cancer biomarker as high USP17 levels are associated with increased rate of disease recurrence and distant metastases.

USPs are emerging as attractive drug discovery targets due to the increasing amount of cancer target validation data alongside their potential druggability on account of their cysteine protease activity [2, 26, 27]. Advances have been made in the development of small molecule inhibitors targeting USPs including USP14, USP9x and USP8. Recently a compound has been developed which selectively inhibits the deubiquitinating activity of USP7 resulting in the stabilisation of p53 and increased apoptosis which may prove important for the treatment of aggressive prostate cancer which is associated with high USP7 levels [9, 28]. The development of anti-DUB therapies is still in its infancy and as yet has not entered clinical trials. However the impact of DUBs to drug discovery has been compared to that of protein kinases and this emerging field will be further fuelled by ongoing advances highlighting the role of DUBs in cancer [29].

Previous shRNA studies have demonstrated that USP17 knockdown reduces proliferation by inhibiting the G1-S phase cell cycle progression and also inhibits chemotaxis [20, 21]. Therefore, in addition to the role we have described for USP17 as a novel cancer biomarker, this DUB could also prove to be a promising anti – cancer therapeutic target. With the need to develop novel pharmacological agents for the treatment NSCLC, USP17 may be a relevant therapeutic target for the treatment NSCLC patients with USP17 positive tumors [30, 31]. This is the first study to directly assess the clinical significance of USP17. These findings demonstrated that NSCLC patients that were positive for USP17 had a significantly reduced recurrence-free survival, marked by metastasis to secondary sites. Much worked is needed to clarify the potentially significant role of USP17 as a cancer biomarker in NSCLC and indeed the involvement of USP17 in metastasis.

## MATERIALS AND METHODS

### NSCLC cohort & Tissue Microarray Construction

NSCLC Tissue Microarrays (TMAs) were constructed using 0.6mm cores in triplicate or quadruplicate, with matched normal representation. Full face H&E sections of each tumor on the TMAs were gigapixel scanned and screened by a consultant histopathologist. Core selections were digitally annotated providing reference to the original location of each TMA core using a web based portal (PathXL™). The catalogue of biopsy tissue comprised a total of 100 individual non-small cell lung cancers that were resected from patients with stage IA to IV disease. The histological subtype of the tumors were either squamous cell carcinoma or adenocarcinoma, and included ever and never smokers. Patients were predominantly treated without neoadjuvant therapy and extensive clinical data was linked. Ethical approval was been granted for collection of archival lung cancer specimens for research, the generation of TMAs (06/NIR01/94).

### Immunohistochemistry and Histological Scoring

Immunohistochemical staining was carried out as described previously [32]. Briefly, 3 μm sections were cut from TMAs and deparaffinised by sequential incubations in xylene and rehydrated through descending grades of alcohol. Endogenous peroxide activity was blocked by incubating with 3% hydrogen peroxide for 10 min and subjected to antigen retrieval (Tris EDTA buffer, pH 9.0 in a pressure cooker for 3 min at 13lb p.s.i./89.6 kPa). USP17 staining of tissue microarrays was carried out as previously described using 2 ug/ml USP17 monoclonal antibody (Fusion Antibodies) followed by application of anti-mouse secondary detection kit (DAKO EnVision HRP labelled polymer) and treatment with 3,3'-diaminobenzidine (DAKO) [20]. Sections were then counterstained with haematoxylin. Immunostained slides were independently scored twice based on the Allred scoring system [33]. Briefly, a proportion score was assigned that represents the estimated fraction of positive tumor cells (range 0-3). The average cytoplasmic staining intensity of positive tumor cells was also noted as being weak, moderate or strong and assigned intensity score (range 1-3). The proportion and intensity scores were then added together to obtain a total histological score (either 0 or ranging 2-6). The average of these scores was taken from replicate TMA cores to obtain a mean total histological score for each patient. Cases were classified as negative (0<2) or positive for USP17 expression (2≤6).

### Statistical Tests

In brief, t tests and one way ANOVA were used to examine the correlation of USP17 with the linked clinicopathological data. Cox's proportional hazards model and Kaplan-Meier survival analyzes were applied to assess the relationship between USP17 and survival and recurrence-free survival. Statistical significance was defined as p<0.05.
